# Diversity and distribution of the cladocerans (Crustacea, Branchiopoda) in Thailand

**DOI:** 10.3897/BDJ.11.e103553

**Published:** 2023-05-25

**Authors:** Wijittra Choedchim, Supiyanit Maiphae

**Affiliations:** 1 Faculty of Science and Technology, Princess of Naradhiwas University, Narathiwat Province, Thailand Faculty of Science and Technology, Princess of Naradhiwas University Narathiwat Province Thailand; 2 Animal Systematics and Ecology Speciality Research Unit (ASESRU), Department of Zoology, Faculty of Science and Biodiversity Center (BDCKU), Kasetsart University, Bangkok, Thailand Animal Systematics and Ecology Speciality Research Unit (ASESRU), Department of Zoology, Faculty of Science and Biodiversity Center (BDCKU), Kasetsart University Bangkok Thailand

**Keywords:** Anomopoda, Ctenopoda, taxonomy, biogeography, Oriental Region

## Abstract

An updated checklist of the cladoceran fauna from inland aquatic habitats in Thailand (a high-diversity hotspot in Southeast Asia), based on published cladoceran records found in literature is presented. The checklist updates nomenclature and species distributions, especially habitat preferences. A total of 138 valid recorded species is relatively high. However, the estimators indicate that more species are expected to be found with more research. The north-eastern and southern regions of Thailand are well-studied regions of high species richness with 100 and 96 cladoceran species, respectively, whereas the northern and eastern regions have large research gaps that should be studied further. Moreover, each habitat type seems to have a unique cladoceran community as the similarity values amongst them are mostly low (Sorensen similarity index < 0.50). Therefore, it is suggested that habitats with unique characteristics, such as peat swamps, stream and cave pools, are worthy of further exploration. If the current records of cladoceran diversity in Thailand confirms a high diversity of this animal in the tropical region, then the geographical distribution of each species can be properly explained.

## Introduction

Thailand is a biodiversity hotspot in Southeast Asia. Few species of freshwater zooplankton have been reported previously, but high diversity is currently shown in various groups, including copepods, rotifers and cladocerans. Research in Thailand on cladocerans began with Boonsom in 1984 and researchers have started to pay more attention to this group of zooplankton since 1997, with more research published. A total of 34 research papers and five research reports have been published, with the majority of studies covering taxonomy and diversity (Boonsom 1984, Pholpunthin 1997, Sanoamuang 1998, Korovchinsky 2000, Pipatcharoenchai 2001, Saeng-aroon 2001, Sa-ardrit 2002, Kotov and Sanoamuang 2004, Kotov et al. 2005a, Kotov et al. 2005b, Maiphae 2005, Maiphae et al. 2005, Sa-ardrit and Beamish 2005, Sanoamuang and Faitakum 2005, Sinev et al. 2007, Sinev and Sanoamuang 2007, Korovchinsky and Sanoamuang 2008a, Maiphae et al. 2008, Chittapun et al. 2009, Maiphae and Janpriang 2009, Maiphae et al. 2010, Choedchim and Maiphae 2012, Meksuwan et al. 2012, Sinev and Kotov 2012, Korovchinsky and Sanomuang 2013, Sinev and Sanoamuang 2013, Van Damme and Maiphae 2013, Van Damme et al. 2013, Tiang-nga et al. 2016, Sinev et al. 2017, Alonso et al. 2019, Jantawong and Maiphae 2020, Tiang-nga et al. 2020, Plangklang and Athibai 2021, Tiang-nga et al. 2021, Sinev et al. 2023) and many fewer covering ecology and aquaculture (Kotov et al. 2013a, Choedchim et al. 2017, Manklinniam et al. 2018).

After almost four decades of intensive study on the diversity of cladoceran in bodies of water in Thailand, 138 species have been identified. However, the taxonomic status of some recorded species has changed given that the taxonomical ranking of these species has changed greatly due to enhanced understanding of their evolution, along with the application of more tools (Van Damme et al. 2005, Van Damme et al. 2010, Van Damme et al. 2011). In addition, it seems that many more species have been discovered in recent years despite its being a relatively well-studied region. This is probably due to the high diversity of microhabitats, with some yet to be surveyed. Therefore, we revise and update the checklist of the cladocerans in Thailand in this paper in light of recent insights into their taxonomy and nomenclature, including analysis of the species diversity and the ecological and geographical distribution of this group. This research contributes to existing knowledge on this important component of freshwater biota in Thailand and offers suggestions for how this knowledge gap could be filled in the future.

## Materials and method

In the present study, a checklist of cladoceran species in Thailand was compiled from the existing 39 research papers and research reports, as mentioned above. The updated names of each species were presented and used for all analyses and the species names used in previous publications were provided. Data on biogeographical distribution are mostly drawn from literature, as shown in Table 1. Occurrences are identified in eight large biogeographical regions (Palearctic, Afrotropical, Oriental, Nearctic, Neotropical, Australian, Pacific and Antarctic), as described in Segers (2007).

**Figure 1.**
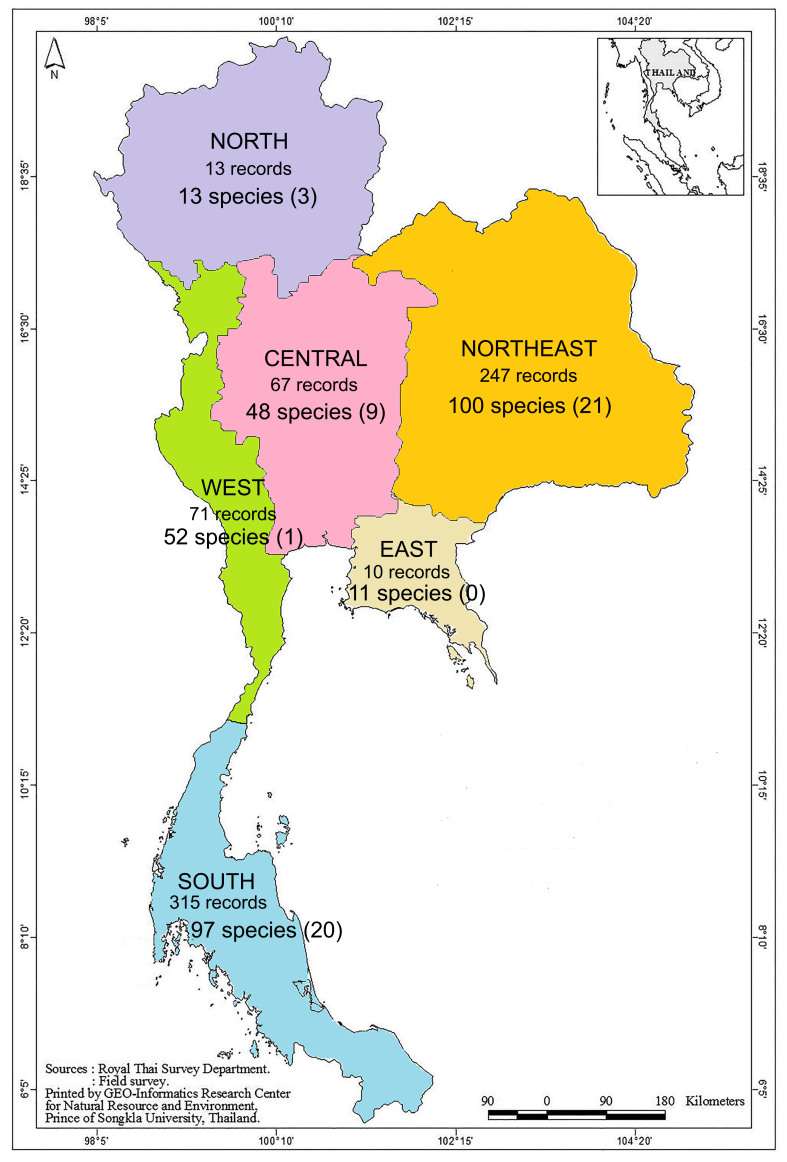
Map of Thailand showing number of data records and species richness found in each region. Numbers in bracket indicate number of species with restricted distribution in that region.

For the data analysis—to answer the following research questions: (1) whether some geographical regions in Thailand were more diverse in cladoceran species than others, regardless of the differences in sampling efforts and (2) whether some habitat types were richer than other types—we divided all the records in Thailand into six geographical regions comprising the north (N), northeast (NE), west (W), east (E), central (C) and south (S) (Fig. 1), based on natural drainage, including landforms and drainage. Northern Thailand is a mountainous area where high mountains are incised by steep river valleys and upland areas that border the central plain. Like the north, the geography of the western region is characterised by high mountains and steep river valleys. The northeast region is a large plateau basin that is extremely flat in some parts with a few low, rugged and rocky hills. Unlike the other areas, the northeast has a long dry season. The central region is a large lowland basin formed by the accumulation of sediment, sand, rocks and mud. The geography of the eastern region is characterised by short mountain ranges alternating with small basins of short rivers that drain into the Gulf of Thailand. Southern Thailand, part of a narrow peninsula, is distinctive in terms of climate, terrain and resources. We also categorised all habitats into 22 types comprising canal, dam, estuary, floodplain, fish field, lake, marsh, mine, man-made lake, pond, pool, peat swamp, river, roadside canal, reservoir, rice field, saline rice field, stream, swamp, temporary pond, wastewater treatment pond and waterfall. The definitions for each habitat type are noted in Suppl. material 1. The species richness estimators, including jackknife1, jackknife2 and bootstrap, were analysed by a species accumulation curve using the EstimateS programme. The jackknife estimator is suitable and tends to reduce the bias in small data samples. In addition, bootstrap is a simple method used to derive estimates of standard errors and confidence intervals for complex estimators of the distribution. Therefore, both estimators were analysed to confirm that the trends in the evaluation results were consistent. In addition, Sorensen’s Similarity Index, which is a statistic used to gauge the similarity of two samples, was used to explore the similarities in species composition amongst regions and habitat types. The index was calculated with Microsoft Excel 2016.

## Results

The dataset contained 723 records for cladoceran published in 39 papers. Overall, seven families, 49 genera and 138 species of cladocerans have been found in Thailand. Of these, 15 species were described from Thailand, while eight were reasoned to be endemic to Thailand. The highest represented families were Chydoridae (80 species), followed by Sididae (18 species), Macrothricidae (16 species), Daphniidae (11 species), Ilyocryptidae (5 species), Bosminidae (4 species) and Moinidae (4 species) (Table 1). The NE region showed the most diverse range of species (100), followed by the S (97), W (52), C (48), N (13) and E regions (11) (Fig. 1).

**Figure 2.**
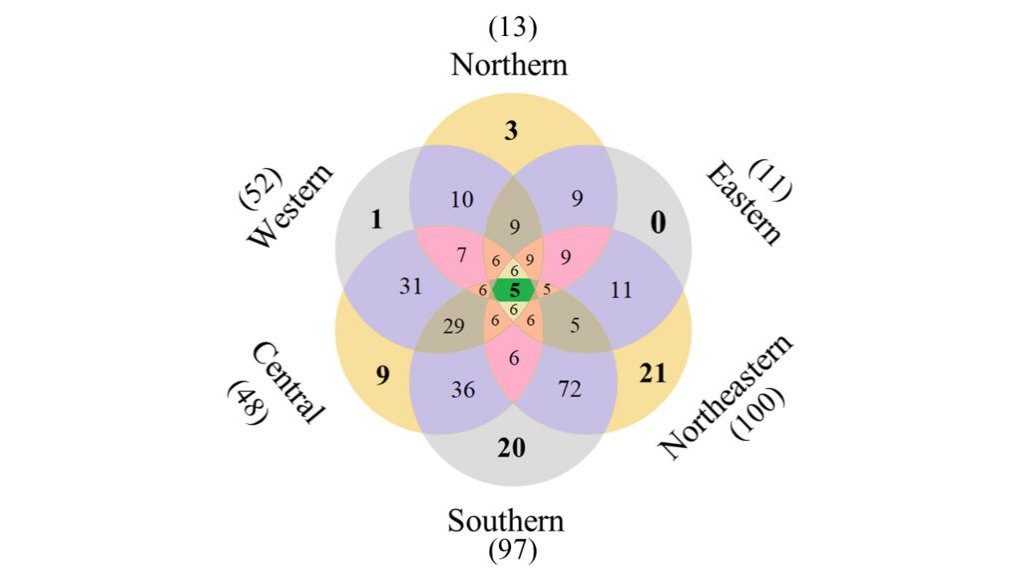
The Venn diagram shows the number of cladocerans restricted to each region and shared between regions. Number in bracket represents number of total species in that region.

Only five species (3.62%)—*Diaphanosomaexcisum*, *D.sarsi*, *D.volzi*, *Latonopsisaustralis* and *Moinasiamensis*— were found in all regions and many species were restricted to only one region. One species, *Bosminafatalis*, has been found only in the W region, while three species (2.17%) have been found only in the N region (Ilyocryptuscf.bhardwaji, *I.raridentatus* and *I.thailandensis*) and nine species (6.52%) have been found only in the C region (*Leberisdavidi*, *Pleuroxusaduncus*, *P.denticulatus*, *Ceriodaphniapulchella*, *C.reticulata*, *Daphniasimilis*, Diaphanosomacf.modigliani, *Macrothrixhirsuticornis* and *Moinamacrocopa*). In the S region, only 20 species (14.49%) have been found (*Alonakotovi*, *Chydorusopacus*, *Ephemeroporusepiaphantoii*, *E.hybridus*, *E.phintonicus*, *E.tridentatus*, *Karualonaiberica*, *K.serrulata*, *Leydigiaaustralis*, *Matralonafreyi*, *Notoalonapseudomacronyx*, *Ovalonaarcheri*, *Pleuroxusuncinatus*, *Salinalonasarasinorum*, Macrothrixcf.gauthieri, *M.malaysiensis*, M.cf.superaculeata, *Diaphanosomacelebensis*, *Sarsilatonapapuana* and *S.serricauda*), while 21 species (15.22%) have been found only in the NE region (*Acroperusafricanus*, *Alonasiamensis*, *Anthalonamilleri*, *A.spinifera*, *Armatoalonamacrocopa*, *Chydorusidrisi*, *C.sinensis*, *Coronatellaacuticostata*, *Disparalonachappuisi*, *Flavalonacostata*, *Karualonaarcana*, *K.kwangsiensis*, *Kurziabrevilabris*, *Leydigialaevis*, *Rheoalonamekongensis*, *Simocephalusexspinosus*, *Ilyocryptusisanensis*, Strebloceruscf.serricaudatus, *S.spinulatus*, *Diaphanosomamacrophthalma* and *D.tropicum*) (Fig. 2).

**Figure 3.**
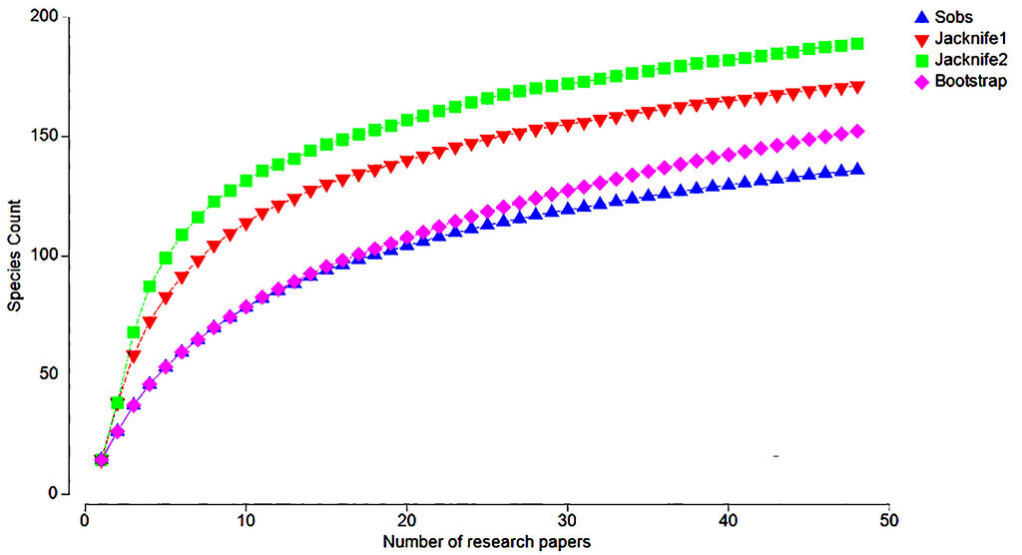
General species accumulation curve over the number of research papers.

According to the general species accumulation curve, the sampling effort (in this case, the number of research papers) is considered insufficient given that the observed values of *S* (138) align with those calculated in the bootstrap estimator (152.94) and the asymptote estimates of the jackknife 1 (170.31) and jackknife 2 (183.20) variation indicators (Fig. 3).

Sorensen’s Similarity Index indicated that the E and N regions showed the highest similarity in terms of cladoceran species composition (0.75), followed by the S and the NE (0.72) and the W and the NE (0.64), whereas the least similarity was found between the S and the N, which were of equal value, along with the S and the E (0.11) (Suppl. material 2).

**Figure 4.**
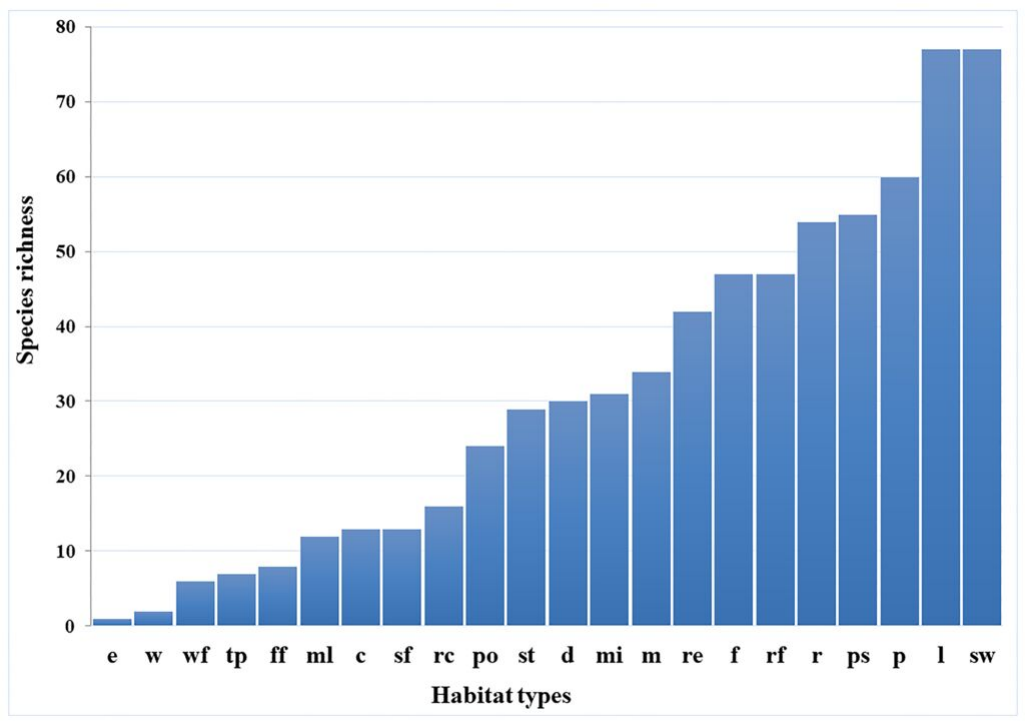
Cladocerans species richness in each habitat type. Abbreviation codes; see Table 1.

The highest species richness was found in swamps and lakes (77 species each), followed by ponds (60 species), peat swamps (55) and rivers (54 species each), whereas estuaries showed the lowest species richness (one species) (Fig. 4). Sorensen’s Similarity Index showed that the similarity of cladoceran species composition was less than 0.50 between most habitat types, whereas only 45 pairs from 231 pairs of different habitats showed a similarity of more than 0.50. Pools and dams had the highest similarity of cladoceran species composition (0.81), followed by mines and dams (0.75), swamps and peat swamps (0.72) and pools and mines (0.69), whereas no similarities (0) were found in 28 pairs of different habitat types (Suppl. material 3).

Twenty-four species were found in various habitat types (> 10 habitats); *Ephemeroporusbarroisi*, *Dunhevediacrassa* and *Ilyocryptusspinifer* occurred in most habitat types (16). In contrast, 28 species were found in only one habitat type: eight species were found only in lakes (*Acroperusafricanus*, *Alonakotovi*, *Chydorusidrisi*, *Coronatellaacuticostata*, *Disparalonachappuisi*, *Flavalonacostata*, *Streblocerusspinulatus* and *Diaphanosomacelebensis*); four species were found only in reservoirs (*Chydorussphaericus*, *Ceriodaphniapulchella*, *C.reticulata* and *Daphniasimilis*); four species were found only in rivers (*Ephemeroporusepiaphantoii*, *Pleuroxusaduncus*, *Rheoalonamekongensis* and *Macrothrixhirsuticornis*); three species were found only in swamps (*Ephemeroporushybridus*, *Notoalonapseudomacronyx* and *Ovalonaarcheri*); two species were found only in ponds (*Anthalonamilleri* and *Leydigialaevis*), two species were found only in marshes (Macrothrixcf.superaculeata and *Pleuroxusuncinatus*) and one species was found only in rice fields (*Karualonaarcana*), floodplains (*Ilyocryptusraridentatus*), peat swamps (*Ilyocryptusthailandensis*), streams (*Diaphanosomatropicum*) and fish fields (*Pleuroxusdenticulatus*). Estuarine habitats mostly showed little or no similarity to other habitats (0–0.03). Only *Salinalonasarasinorum* could be found in estuarine waters at a distribution of up to 12 part per thousand.

## Discussion

### Species richness

Since being poorly known in Thailand 30 years ago, the number of identified and studied cladoceran species has continued to increase. More intensive diversity studies in various types of microhabitats, including the taxonomic revision of some species, have led to more species being recorded. A total of 38 new records have been identified during the past 15 years compared to the records of Maiphae et al. (2008). Of these, 15 species are described from Thailand and eight species are endemic to Thailand. In addition, 16 synonymies were detected in previous records (Table 1). The total number of species identified in Thailand is relatively high and accounts for approximately 45% (about 298 species) of all records in Southeast Asia (Korovchinsky 2013, Tiang-nga et al. 2020). In addition, the species richness of cladocerans in Thailand is relatively high compared with records from other countries in Southeast Asia, as shown by the following statistics: Malaysia has about 104 species (Korovchinsky 2013, Sinev and Yusoff 2015); Indonesia has about 118 species (Korovchinsky 2013); the Philippines has about 55 species (Korovchinsky 2013, Pascual et al. 2014,Lopez et al. 2017); Laos has about 80 species (Kotov et al. 2013b, Siboualipha et al. 2020); Cambodia has about 60 species (Tanaka and Ohtaka 2009); Vietnam has about 130 species (Sinev and Korovchinsky 2013, Korovchinsky 2013, Sinev 2014, Sinev and Irina 2021). These differences are not only because more sampling sites were explored in Thailand, but also because the studied sites included a high diversity of habitat types (22 types). However, the estimator indices indicated that the present number of records is an underestimate and that more species could be discovered in Thailand with more research, particularly in less studied regions (i.e. the N, W and E). Currently, high species richness is found in the W, despite relatively few sites being sampled in comparison to the size of the area. The W region comprises mountain ranges and plains and is similar to the N region. Therefore, it would be interesting to explore more sites and microhabitats, especially peat swamps, streams and cave pools, as the discovery of more species is expected.

The N region of Thailand is relatively large. However, few studies have been conducted despite all cladoceran microhabitats being represented. Researchers have focused on the Ilyocryptidae (Kotov and Sanoamuang 2004), Sididae (Korovchinsky and Sanomuang 2013) and Moinidae (Alonso et al. 2019) families following their interest in taxa reported from the N. Likewise, only the Sididae and Moinidae families (Alonso et al. 2019) have been researched in the E region. This is one reason why the cladoceran compositions in these two regions have a high level of similarity. Likewise, the NE and S regions also show numerous similarities in cladoceran composition due to a similar research focus. The E region is the smallest in Thailand, but it is the most interesting to investigate due to its diverse geography (river basins and coastal areas with a mountain range in the middle). However, the N and E regions have a large research gap that could be targeted by further studies researching their species diversity. The distribution pattern for the species and range boundaries of each species could then be tentatively outlined and more extensive zoogeography could be analysed.

### Geographical distribution

Present records show that the proportion of commonly distributed species is less than that of restricted species. Only *Diaphanosomaexcisum*, *D.sarsi*, *D.volzi*, *Latonopsisaustralis* and *Moinasiamensis* were found in all regions. Of the other species, *Bosminopsisdeitersi* and *Ephemeroporusbarroisi* are also common, as they are distributed in every region, except the N, which might be because studies are lacking in that region, as mentioned previously. Korovchinsky (1992) made it clear that Sididae, especially the genus *Diaphanosoma*, contribute substantially to all continents. In addition, amongst Moinidae, *Moina* is much more common in the limnetic zone of tropical lakes. These small and transparent species are relatively immune to fish predation (Dumont 1994), which could explain their wide distribution, especially in oriental and circumtropical regions that have a high abundance of planktivorous fish. *Bosminopsisdeitersi* is a species known for its multicontinental range and broad ecological requirements (Garibian et al. 2021). The hidden diversity of this species would be interesting to investigate. *Moinasiamensis* was recently described in Thailand (Alonso et al. 2019), where it is found in every region. Existing records for *M.siamensis* in Thailand need to be re-examined, however, because its characteristics are similar to *M.micrura* (another widely distributed species in Thailand). Notably, *Daphnia* is almost absent from the country; of this wide range of environmentally tolerant species, only *D.lumholtzi* can be found. This result differs from tropical India, which has a relatively high diversity of Daphnia (Ctenodaphnia) (Padhye et al. 2016). Besides latitude, which positively correlates to the distribution of this genus, lower temperature (compared to Thailand), even in its tropical zone, might be the reason for the higher richness of this genus in India. However, other factors, such as predators, might co-influence the distribution of this genus and other planktonic ones. The genus rarely found in Thailand is replaced by more Sidids, Moinids and Bosminids, as mentioned previously. In tropical regions, fish are more numerous than elsewhere and it is hypothesised that the effects of predation by planktivorous fish are high here. Usually, large *Daphnia* cannot survive under intensive fish predation. Additionally, the tropics also contain invertebrates that are known to prey on *Daphnia*, such as the larvae of the phantom midge *Chaoborus* and the water boatman *Notonecta* (Ebert 2005).

### Microhabitat distribution

Lakes and swamps are heterogeneous environments that harbour the highest cladoceran diversity and include high-richness habitats. A total of 77 species are found in these habitats, accounting for about 56% of the known cladoceran species in Thailand. Two biologically rich lakes in Thailand, Kud-Thing Lake and Thale-Noi Lake, are Ramsar sites where fauna thrive. Apart from cladocerans, other groups of zooplankton, fish, birds and aquatic plants have high diversity in these lakes. Kud-Thing Lake is a large natural lake connected to the Mekong River and Thale-Noi Lake is connected to Songkhla Lake (Ramsar Sites Information Service 2023). These geographical characteristics provide complex lake structures that enable organisms to live in several microhabitats and ecological niches.

It was also found that similar habitat structures led to similar cladoceran compositions. Pools, dams and mines are permanent man-made habitats that show a high similarity of cladoceran compositions. Swamps and peat swamps, which are natural habitats mostly covered with aquatic plants, also showed high similarity in cladoceran composition. Some types of habitats, such as estuarine waters, have unique structures, leading to low similarity with other habitats. The species found in these unique habitats, such as *Salinalonasarasinorum*, warrant further study, particularly in other research fields, such as ecophysiology. Some cladoceran habitats have scarcely been studied, including peat swamps, streams and cave pools. Thailand has several small and large cave systems in each region. Copepods are a good example of organisms that are well studied in cave pools and high numbers of copepods are seen in this harsh habitat (Watiroyram 2021, Sanoamuang and Watiroyram 2021). It is expected that some yet-to-be-discovered cladoceran species may be present.

Although the taxonomy and distribution of most cladoceran species are now clearly understood, further studies should be carried out to reach a plateau. To determine the actual species richness of the country and gain a greater understanding of the ecological and biogeographical distribution of cladocerans, increased sampling efforts should be directed at less-studied habitats, such as peat swamps, streams, cave pools and groundwater. In addition, the habitats on islands in the Thai–Malaysia Peninsula would also be interesting to explore and are anticipated to contribute greatly to a better understanding of the biogeographical distribution of this animal in Southeast Asia. Moreover, it would be interesting to further integrate both morphological and genetic diversity given that cryptic species are assumed to be widely distributed in nature and amongst biogeographical regions (Pfenninger and Schwenk 2007) and that their discovery and description are pivotal to the correct assessment of actual biodiversity patterns. Since we now know that the cladoceran community in Thailand could somehow be representative of tropical countries, it would be interesting to use the cladoceran species as a model to study functional traits and as bioindicators to measure the health of aquatic environments. This would meet the purpose of this updated checklist, which aims to contribute to more aspects of cladoceran research in tropical regions.

## Supplementary Material

0F024115-EB07-5C9F-BA2F-68A5FD9B6194 10.3897/BDJ.11.e103553.suppl1 Supplementary material 1 Definitions of each type of habitat in this study Data type definition Brief description This document describes the features used to identify each type of water source in this study. File: oo_845357.docx https://binary.pensoft.net/file/845357 Wijittra Cheodchim and Supiyanit Maiphae

EB03E2CF-E9B8-503A-BF84-FB3952CBC9F1 10.3897/BDJ.11.e103553.suppl2 Supplementary material 2 Sorensen’s Similarity Index of cladoceran species amongst geographical regions Data type index File: oo_846715.xlsx https://binary.pensoft.net/file/846715 Wijittra Cheodchim and Supiyanit Maiphae

60FE59A0-9259-58A5-9146-2CF24D1F3B8A 10.3897/BDJ.11.e103553.suppl3 Supplementary material 3 Sorensen’s Similarity Index of cladoceran species amongst habitat types Data type index File: oo_846716.xlsx https://binary.pensoft.net/file/846716 Wijittra Choedchim and Supiyanit Maiphae

## Figures and Tables

**Table 1.** List of cladoceran species, their habitat occurrence and distribution in Thailand. (Abbreviation codes: c = canal, d = dam, e = estuary, f = floodplain, ff = fish field, l = lake, m = marsh, mi = mine, ml = man-made lake, p = pond, po = pool, ps = peat swamp, r = river, rc = roadside canal, re = reservoir, rf = rice field, sf = saline rice field, st = stream, sw = swamp, tp = temporary pond, w = wastewater treatment pond, wf = waterfall, N = north, NE = northeast, W = west, E = east, C = central, S = south, Aus = Australian, Afr = Afrotropical, Nea = Nearctic, Neo = Neotropical, Ori = Oriental, Pal = Palearctic; Reference codes: 1 = Boonsom 1984, 2 = Pholpunthin 1997, 3 = Sanoamuang 1998, 4 = Saeng-aroon 2001, 5 = Pipatcharoenchai 2001, 6 = Sa-ardrit 2002, 7 = Kotov and Sanoamuang 2004, 8 = Sa-ardrit and Beamish 2005, 9 = Kotov et al. 2005a, 10 = Kotov et al. 2005b, 11 = Maiphae 2005, 12 = Maiphae et al. 2005, 13 = Sanoamuang and Faitacum 2005, 14 = Sinev et al 2007, 15 = Sinev and Sanoamuang 2007, 16 = Maiphae et al. 2008, 17 = Korovchinshky and Sanoamuang 2008a, 18 = Chittapun et al. 2009, 19 = Maiphae and Janpriang 2009, 20 = Maiphae et. al. 2010, 21 = Meksuwan et al. 2012, 22 = Choedchim and Maiphae 2012, 23 = Sinev and Kotov 2012, 24 = Kotov et al. 2013a, 25 = Van Damme and Maiphae, 2013, 26 = Van Damme et al. 2013, 27 = Sinev and Sanoamuang 2013, 28 = Korovchinsky and Sanoamuang 2013, 29 = Tiang-nga et al. 2016, 30 = Choedchim et al. 2017, 31 = Sinev et al. 2017, 32 = Manklinniam et al. 2018, 33 = Alonso et al. 2019, 34 = Jantawong and Maiphae 2020, 35 = Korovchinsky 2000, 36 = Tiang-nga et al. 2020, 37 = Tiang-nga et al. 2021, 38 = Plangklang and Athibai 2021, 39 = Sinev et al. 2023).

	Species	Habitat occurrence	Distribution in Thailand	Biogeographical distribution	Remarks	References for records in Thailand
	Family Bosminidae					
1	*Bosminafatalis* Burckhardt, 1924	sw, ml, r, re	W	Ori	It is rare in Thailand.	5
2	*Bosminalongirostris* (O. F. Müller, 1776)	sw, re	W, C, S	Cosmopolitan	It can be confused with *B.fatalis* (Kořínek et al. 1997).	1,5,11,12
3	*Bosminameridionalis* Sars, 1904	f, l, p, ps, r, re, rf, sw	NE, S	Aus, Ori		3,13,19,30,36
4	*Bosminopsisdeitersi* Richard, 1895	d, f, l, mi, ml, p, po, r, rc, re, rf, st, sw, tp, wf	NE, W, E, C, S	Cosmopolitan		1,2,3,4,5,6,8,11,12,13,20,30,36
	Family Chydoridae					
5	*Acroperusafricanus*Neretina & Kotov, 2015	l	NE	Aus, Ori	It can be confused with *A harpae* and *A.angustatus* (Neretina and Kotov 2015).	36
6	*Acroperusharpae* (Baird, 1834)	c, m, ps, r, sw	NE, S	Cosmopolitan (widely distributes in Pal)	It can be confused with sibling species, *A.angustatus* (Sinev 2009).	3,4,6,11,12
7	*Alonaaffinis* (Leydig, 1860)	f, l, m, mi, p, ps, r, rf, st, sw	NE, W, C, S	Afr, Aus, Neo, Ori, Pal		1,3,4,6,8,11,12,13,19,36
8	*Alonaguttata* Sars, 1862	d, f, l, m, p, po, ps, r, mi, st, sw	NE, W, S	Cosmopolitan		4,6,8,11,12,13,30,36
9	*Alonaintermedia* Sars, 1862	l, sw	NE, S	Cosmopolitan		4,6
10	*Alonakotovi* Sinev, 2012	l	S	Ori	Known from South Vietnam and it is rare in Thailand.	30
11	*Alonaquadrangularis* (O.F. Müller, 1776)	f, l, m, p, r	NE, S	Afr, Aus, Neo, Ori	Sinev (2012) proposed that previous records of *A.quadrangularis* from Southeast Asia (Korovchinsky 2013) belong to *A.kotovi* from South Vietnam.	4,11,12,13
12	*Alonasiamensis* Sinev & Sanoamuang, 2007*	f, ps	NE	Ori	Previously recorded as *Alonacf.dentifera* (Maiphae 2005, [Bibr B9194126][Bibr B9194387], Maiphae et al. 2005, Sanoamuang and Faitakum 2005, Nachai 2006).	15
13	*Alonellaclathratula* Sars, 1896	f, l, p, r, sw	NE, S	Afr, Aus, Neo, Ori	It is rare in Thailand.	3,4,11,12,13
14	*Alonellaexcisa* (Fischer, 1854)	c, d, f, l, mi, p, po, ps, re, sw,	NE, S	Cosmopolitan		2,3,4,6,11,12,13,19,36
15	*Alonellanana* (Baird, 1850)	m, ps, r, re, sw	C, S	Afr, Aus, Pal	It is rare in Thailand.	1,6,11,12,30
16	*Anthalonaharti* Van Damme, Sinev & Dumont, 2011	l, p, ps, r, rf, sf, sw	NE, C, S	Afr, Ori	It is a sibling species of *Alonaverrucosa* (Sinev and Kotov 2012).	30,34,36,38
17	*Anthalonamilleri* (Kiser, 1948)	p	NE	Ori	It is rare in Thailand. Previously recorded as *Alonamilleri* (Sanoamuang 1998).	3
18	*Anthalonasanoamuangae* Sinev & Kotov, 2012*	l, r	NE, S	Ori	Known from Vietnam, Laos and it is rare in Thailand.	23,30
19	*Anthalonaspinifera* Tiang-nga, Sinev & Sanoamuang, 2016*	l, r, rf, sw	NE	Ori	Known from Malaysia and it is rare in Thailand.	29,36
20	*Anthalonavandammei* Sinev, Tiang-nga & Sanoamuang, 2023*	l, sw	NE, S	Ori	Previously recorded as *Alonaverrucosa* (Maiphae et al. 2008). Endemic in Thailand.	39
21	*Anthalonaverrucosa* (Sars, 1901)	c, d, l, m, mi, p, po, ps, r, re, rf, sw	NE, W, S	Afr, Aus, Neo, Ori	All records as *Alonaverrucosa* in Thailand before Van Damme et al. (2010) needed to be confirmed their species status.	1,3,4,5,6,8,11,12,13,19,20
22	*Armatalonamacrocopa* (Sars, 1894)	l, tp	NE	Aus, Ori	In the Oriental Region, it was known only from Thailand.	14
23	*Camptocercusaustralis* Sars, 1896	f, l, p, po, st, sw	NE, W, S	Aus, Neo, Ori		4,6,8,11,12,13,30
24	*Camptocercusrectirostris* Schoedler, 1862	l, ml	W, C, S	Ori, Pal		1,2
25	*Camptocercusuncinatus* Smirnov, 1971	l, r, re	NE, W, S	Afr, Neo, Ori, Pal	*Camptocercuslatikae* is its junior synonym.	2,3,5
26	*Celsinotummacronyx* (Daday, 1898)	f, l, p, ps, rf, sw	NE, S	Ori	Previously recorded as *Alonamacronyx* (Sanoamuang 1998, Sa-ardrit 2002, Maiphae et al. 2005).	3,6,11,12,23,30
27	*Chydoruseurynotus* Sars, 1901	c, d, f, l, m, mi, p, po, ps, r, re, rf st, sw, wf	NE, W, C, S	Circumtropical		1,2,3,4,5,6,8,11,12,13,19,30,34,36
28	*Chydorusidrisi* Sinev, 2014	l	NE	Ori		36
29	*Chydorusobscurirostris* Frey, 1987	d, p, ps, r, sw,	NE, S	Aus, Ori		6,11,12,13
30	*Chydorusopacus* Frey, 1987	ps, sw	S	Aus, Ori	It is rare in Thailand.	6
31	*Chydorusparvus* Daday, 1898	d, f, l, m, mi, p, po, ps, r, rc, re, rf, st, sw	NE, W, S	Afr, Ori		3,4,5,6,8,11,12,13,19,30
32	*Chydoruspubescens* Sars, 1901	d, l, m, mi, p, po, ps, r, st, sw	NE, W, S	Circumtropical		3,6,8,11,12,13,36
33	*Chydorusreticulatus* Daday, 1898	d, f, l, m, mi, ml, p, ps, r, rf, sw	NE, W, S	Ori		2,3,5,6,11,12,13,19,36
34	*Chydorussinensis* Frey, 1987	f, l, sw	NE	Ori	Closely related to *C.obscurirostristasekberae* (Sanoamuang 1998). It was recorded from China and Thailand.	3,4,13
35	*Chydorussphaericus* (O.F. Müller, 1776)	re	NE, C, S	Cosmopolitan		1,11,12
36	*Chydorusventricosus* Daday, 1898	c, d, f, m, mi, ml, p, po, ps, r, rf, st, sw	NE, W, C, S	Circumtropical		1,3,6,8,11,12,13,19,30,36
37	*Coronatellaacuticostata* (Sars, 1903)	l	NE	Ori	Closely related to *C.undata* (Fuentes-Reinés et al. 2021).	36
38	*Coronatellamonacantha* (Sars, 1901)	d, f, l, m, mi, p, po ps, rf, sw, wf	NE, S	Afr, Neo, Ori	Previously recorded as *Alonamonacantha* (Sanoamuang 1998, Sa-ardrit 2002, Maiphae et al. 2005, Maiphae and Janpriang 2009).	3,6,11,12,13,19,20,30
39	*Coronatellarectangula* (Sars, 1862)	d, l, p, po, ps, r, re, st, sw	NE, W, S	Cosmopolitan	All previous references were recorded as *Alonarectangular*, except Choedchim et al. (2017) and Tiang-nga et al. (2020). *Alonacoronata* is its junior synonym.	2,3,5,6,8,11,12,30,36
40	*Dadayamacrops* (Daday, 1898)	d, f, l, m, mi, p, po, ps, r, rc, rf, st, sw	NE, W, C, S	Circumtropical		1,3,4,6,8,11,12,13,19,20,36
41	*Disparalonacaudata* Smirnov, 1996	r, re, sw	NE, S	Aus, Ori	Closely related to *D.rostrata* (Sanoamuang 1998).	3,11,12
42	*Disparalonachappuisi* Brehm, 1934	l	NE	Afr, Ori, Pal	It is rare in Thailand.	36
43	*Disparalonahamata* Birge, 1879	d, ps, r, sm	NE, W, S	Cosmopolitan		3,4,6,8,11,12,13
44	*Disparalonarostrata* (Koch, 1841)	d, f	NE, S	Ori, Pal	It is rare in Thailand.	6,13
45	*Dunhevediacrassa* King, 1853	d, f, l, m, mi, ml, p, po, ps, r, re, rf, sf, st, sw, w	NE, W, C, S	Cosmopolitan		1,2,3,4,5,6,8,11,12,13,19,24,30, 36,38
46	*Dunhevediaserrata* Daday, 1898	f, l, m, mi, p, ps, rf, sw	NE, W, S	Afr, Ori		3,4,6,8,11,12,13,19,36
47	*Ephemeroporusbarroisi* (Richard, 1894)	c, d, f, l, m, mi, ml, p, po, ps, r, re, rf, sf, st, sw, wf	N, NE, W, C, S	Cosmopolitan		1,2,3,4,5,6,8,11,12,13,19,20,21, 30,34,36,38
48	*Ephemeroporusepiaphantoii* Alonso, 1987	r	S	Pal, Ori	In the Oriental Region, it was known only from Thailand.	21
49	*Ephemeroporushybridus* (Daday, 1905)	sw	S	Afr, Nea, Neo, Ori		11,12
50	*Ephemeroporusphintonicus* (Margaritora, 1969)	m, p, ps, sw	S	Aus, Ori		6,11,12
51	*Ephemeroporustridentatus* (Bergamin, 1939)	ps, re, sw	S	Neo, Ori		11,12
52	*Euryalonaorientalis* (Daday, 1898)	l, p, ps, r, rc, re, rf, st, sw	NE, W, C, S	Circumtropical		1,2,3,4,5,8,11,12,13,18,19,30,36
53	*Flavalonacheni* (Sinev, 1999)	m, rf, sw	C, S	Afr, Ori, Pal	Previously recorded *as Alonacheni* (Maiphae et al. 2005, Maiphae and Janpriang 2009, Chittapun et al. 2009).	11,12,18,19
54	*Flavalonacostata* (Sars, 1862)	l	NE	Afr, Neo, Ori, Pal		36
55	*Graptoleberistestudinaria* (Fischer, 1848)	l, p, sw	NE, S	Cosmopolitan		3,6,36
56	*Karualonaarcana* Tiang-nga, Sinev & Sanoamuang, 2021*	rf	NE	Ori	Endemic in Thailand.	37
57	*Karualonaiberica* (Alonso & Pretus, 1989)	m, ps, re, rf, sw	S	Afr, Aus Ori, Pal		11,12,19,20
58	*Karualonakarua* (King, 1853)	f, l, ml, ps, re, sf, sw	NE, C, S	Aus, Ori, Pal		1,2,13,20,30,34,36,38
59	*Karualonakwangsiensis* (Chiang 1963)	l, r, rf, sw	NE	Ori		36
60	*Karualonaserrulata* Van Damme, Maiphae & Sa-ardrit, 2013*	ps, sw	S	Ori	*Karualona* sp. in Sa-ardrit (2002) represents this species. Endemic in Thailand.	26
61	*Kurziabrevilabris* Rajapaksa & Fernando, 1986	f, l	NE	Ori	Endemic in the Oriental Region.	13,36
62	*Kurzialongirostris* (Daday, 1898)	c, l, m, ml, ff, ps, rc, re, rf, st, sw	NE, W, C, S	Aus, Ori		1,3,4,5,6,8,11,12,18,30,36
63	*Leberisdavidi* (Richard, 1895)	ff, r	C	Neo, Nea, Ori	Previously recorded as *Alonadavidi* (Boonsom 1984).	1
64	*Leberisdiaphanus* (King, 1853)	sf, sw	NE, W, C, S	Afr, Aus, Ori	Previously recorded as *Alonadiaphana* (Sanoamuang 1998, Sa-ardrit 2002, Sa-ardrit and Beamish 2005, Maiphae et al. 2005, Sanoamuang and Faitakum 2005). It is misspelled as *Leberisdiaphana* in Maiphae and Janpriang (2009).	2,3,4,6,8,11,12,13,19,30,34, 36,38
65	*Leydigiaacanthocercoides* (Fischer, 1854)	l, ml, re, st	NE, W, C, S	Pal, Ori		1,3,5,8,30
66	*Leydigiaciliata* Gauthier, 1939	l, ps	NE, S	Afr, Aus Neo, Ori	*L.ankammaraoi* is its junior synonym.	2,13,38
67	*Leydigialaevis* Gurney, 1927	p	NE	Aus, Ori	In the Oriental Region, it was known only from Thailand.	3
68	*Leydigiaaustralis* Sars, 1885	d, l	S	Aus, Ori		6,30
69	*Matralonafreyi* (Idris & Fernando, 1981)	p, ps, sw	S	Ori	Previously recorded as *Alonafreyi* (Sa-ardrit 2002, Maiphae et al. 2005).	8,11,12,13,36
70	*Nicsmirnoviuseximius* (Kiser, 1948)	f, ps, st, sw	NE, W, S	Aus, Ori		8,11,12,13,36
71	*Notoalonaglobulosa* (Daday, 1898)	f, d, c, m, mi, l, p, po, ps, r, rc, rf, sw	NE, S	Afr, Aus, Neo, Ori		3,4,6,11,12,
						13,19,30,36
72	*Notoalonapseudomacronyx* Van Damme, Maiphae & Sa-ardrit, 2013*	sw	S	Afr, Ori		26
73	*Oxyurellasingalensis* (Daday, 1898)	d, f, l, m, mi, p, po, ps, r, rc, re, rf, st	NE, W, C, S	Afr, Ori		1,3,4,6,8,11,12,13,19,20,36
74	*Ovalonaarcheri* Sars, 1888	sw	S	Aus, Ori	Previously recorded as *Alonaarcheri* (Maiphae 2005).	2,11,12,19
75	*Ovalonacambouei* de Guerne & Richard, 1893	l, p, ps, rf, sf	NE, S	Afr, Ori, Pal	Previously recorded as *Alonacambouei* (Maiphae 2005). It can be confused with a sibling species, *O.pulchella* (Maiphae 2014).	3,11,12,19,20,36,38
76	*Ovalonapulchella* King, 1853	l, p, r, rf	NE, C, S	Afr, Neo, Ori	Previously recorded as *Alonapulchella* (Maiphae 2005). It is a species group and it is a sibling species of *O.cambouei* and *O.glabra* (Sinev 2015).	3,4,18,20,34
77	*Pleuroxusaduncus* (Jurine, 1820)	r	C	Cosmopolitan		1
78	*Pleuroxusdenticulatus* Birge, 1879	ff	C	Afr, Nea Pal, Ori		1
79	*Pleuroxusuncinatus* Baird, 1850	m	S	Afr, Aus, Neo, Ori, Pal	It is closely related to *P.trigonellus* and *P.bdatonicus* is its junior synonym (Frey 1965).	11,12
80	*Pleuroxusquasidenticulatus* Smirnov, 1996	st, sw	NE, W, S	Aus, Neo, Ori, Pal	It is closely related to *P.denticulatus* (Sinev and Sanoamuang 2013). In the Oriental Region, it was known only from Thailand.	6,8,27
81	*Picripleuroxuslaevis* (Sars, 1862)	mi, p,	NE, W, S	Afr, Aus Ori, Pal		3,6,8,11,12,13,19
82	*Pseudochydorusglobosus* (Baird, 1843)	f, l	NE, S	Cosmopolitan		13,30
83	*Rheoalonamekongensis* Sinev, Tieng-nga & Sanoamuang, 2017*	r	NE	Ori	Endemic in Thailand.	31
84	*Salinalonasarasinorum* Van Damme & Maiphae, 2013*	e, sw	S	Ori	Previously recorded as *Alonasarasinorum* (Maiphae 2005). *A.taraporevalae* is its junior synonym.	11,12,25
	Family Daphniidae					
85	*Ceriodaphniacornuta* Sars, 1885	f, ff, l, ml, ps, r, re, rf, sf, st, sw, tp	NE, W, C, S	Cosmopolitan		1,3,4,5,6,8,11,12,13,18,19,20,30, 34,36,38
86	*Ceriodaphniapulchella* Sars, 1862	re	C	Afr, Ori	It is rare and the occurrences in Thailand need to be confirmed.	1
87	*Ceriodaphniareticulata* (Jurine, 1820)	re	C	Afr, Neo,Nea, Ori, Pal	It is rare and the occurrences in Thailand need to be confirmed. Its junior synonyms are *C.serrata* and *C.kuerzii*.	1
88	*Daphnialumholtzi* Sars, 1885	f, l, st	NE, W, C	Afr, Aus, Nea, Neo, Ori	*Daphniopsissumanae* Rane, 1986 is its junior synonym.	1,3,4,5,8,13,36
89	*Daphniasimilis* Claus, 1876	re	C	Ori, Pal	Hudec (1991) proposed that *D.similis* in Asia may belong to *D.similoides*. Therefore, the species status needs to be confirmed.	1
90	*Scapholeberiskingi* Sars, 1903	d, f, l, m, mi, ml, p, po, ps, r, re, rf, st, sw	NE, W, C, S	Afr, Aus, Ori, Pal		1,3,4,5,6,8,11,12,13,18,19,20,36
91	*Simocephalusexspinosus* (De Geer, 1778)	f, l, p	NE	Aus, Ori, Pal		3,4,13
92	*Simocephalusheilongjiangensis* Shi & Shi, 1994	d, f, l, m, p, po, ps, rf, st, sw	NE, W, S	Afr, Aus, Ori	Previously recorded as *Simocephalusmesorostris* (Sa-ardrit 2002;Maiphae et al. 2005). *S.mesorostris* is its junior synonym.	3,4,6,8,11,12,13,19,36
93	*Simocephaluslatirostris* Stingelin, 1906	l, r, re	C, S	Aus, Neo, Ori		1,30
94	*Simocephalusvetulus* (O.F. Müller, 1776)	p, r, re, sw	NE, C	Afr, Aus, Neo, Ori, Pal	Closely related to sibling species, *S.mixtus*, *S.vetuloides*, *S.gibbosus*, *S.elizabethae* and *S.punctatus* (Orlova-Bienkowskaja 2001).	1,3
95	*Simocephalusserrulatu*s (Koch, 1841)	d, f, l, mi, p, ps, r, rf, st, sw	NE, W, S	Afr, Aus, Nea, Neo, Ori		3,4,6,8,11,12,13,19,30,36
	Family Ilyocryptidae					
96	Ilyocryptuscf.bhardwaji Battish, 1981	no data	N	Ori	Known from India and Thailand.	7
97	*Ilyocryptusisanensis Kotov*, Stifter & Sanoamuang, 2005*	rf, tp	NE	Ori	Endemic in Thailand.	10
98	*Ilyocryptusraridentatus* Smirnov, 1989	f	N	Aus, Ori	Its junior synonyms are *I.cf.sarsi* in Kotov and Štifter (2006) and I.cf.raridentatus in Kotov et al. (2011), (Kotov et al. 2012).	7
99	*Ilyocryptusspinifer* Herrick, 1882	c, d, f, l, mi, ml, p, po, ps, r, rc, re, rf, sf, st, sw	NE, W, C, S	Cosmopolitan	The junior synonym are *I.agilis* in Kim (1988), *I.sordidus* in Chiang and Du (1979)(Kotov et al. 2012) and *I.halyi* (Michael and Sharma 1988, Kotov and Dumont 2000).	1,3,4,5,6,8,11,12,13,18,19,30, 36,38
100	*Ilyocryptusthailandensis* Kotov & Sanoamuang, 2004*	ps	N	Ori	Endemic in Thailand.	7
	Family Macrothricidae					
101	*Grimaldinabrazzai* Richard, 1892	f,l, p, re	NE, W, S	Circumtropical		5,6,13,30,36
102	*Guernellaraphaelis* Richard, 1892	f, l, p, r, rc, rf, sw	NE, W, C, S	Circumtropical		3,6,8,11,12,13,18,19,20,30,36,38
103	*Macrothrixflabelligera* Smirnov, 1992	c, d, l, m, mi, p, ps, po, r, sw, wf	NE, S	Aus, Ori	In the Oriental Region, it was known from Thailand and Cambodia.	2
104	Macrothrixcf.gauthieri Smirnov, 1976	m, r	S	Afr, Aus, Ori	It can be confused with *M.triserialis* (Smirnov 1992).	11,12
105	*Macrothrixhirsuticornis* Norman & Brady, 1867	r	C	Pal	Kotov (2007), Kotov (2008) confirms its distribution only in Pal. Therefore, the occurrence in Thailand needs to be confirmed.	1
106	Macrothrixcf.laticornis (Fischer, 1851)	l, ml, p, r, rf, st, sw, tp	NE, W, S	Aus, Neo, Ori, Pal	*M.bialatusi*s its junior synonym. In the Oriental Region, it was known only from Thailand.	3,5,6,8,11,12
107	*Macrothrixmalaysiensis* Idris & Fernando, 1981	ps, sw	S	Aus, Ori		11,12
108	*Macrothrixodiosa* Gurney, 1916	d, f, l, mi, p, ps, rf, sw	NE, S	Afr, Aus Ori, Pal		4,6,11,12,13,19,30,36
109	*Macrothrixpaulensis* (Sars, 1900)	m, p	NE, S	Neo, Ori	It is rare in Thailand.	3,11,12
110	*Macrothrixpholpunthini* Kotov, Maiphae & Sanoamuang, 2005*	l, ps	NE, S	Ori	It was known from Thailand and Cambodia.	9,26,30,36
111	*Macrothrixspinosa* King, 1853	d, f, l, m, mi, p, po, ps, r, rc, re, rf, sf, st sw	NE, W, C, S		*Macrothrixgoeldi* is its junior synonym.	2,3,4,6,8,11,12,13,18,19,20,30, 34,36,38
112	Macrothrixcf.superaculeata Smirnov, 1982	m	S	Neo, Ori	It is rare in Thailand.	11,12
113	*Macrothrixtriserialis* Brady, 1886	d, f, l, m, p, po, ps r, rc, re, rf, sf, sw	NE, W, C, S	Circumtropical		1,2,3,5,6,8,11,12,13,19,20,30, 36,38
114	*Strebloceruspygmaeus* Sars, 1901	-mi, p, re, sw, wf	NE, W, C, S	Neo, Ori		1,3,5,6,11,12
115	Strebloceruscf.serricaudatus (Fisher 1849)	l, re	NE	Aus, Nea, Ori Pal		36
116	*Streblocerusspinulatus* Smirnov, 1992	l	NE	Ori	Endemic in the Oriental Region.	36
	Family Moinidae					
117	*Moinamacrocopa* (Straus, 1820)	ff, rf	C	Ori, Pal	Its junior synonyms are *M.easu* and *M.ganapati*.	1,32
118	*Moinamicrura* Kurz, 1874	f, l, p, r, rc, re, rf, sw, w, tp	NE, W, C, S	Cosmopolitan	*M.dodhui* is its junior synonym.	1,3,4,5,6,8,11,12,13,20,30,34, 36,38
119	*Moinasiamensis* Alonso, Neretina, Sanoamuang, Saengphans & Kotov, 2019*	po, rc, rf, w	N, NE, W, E, C, S	Ori	It could be easily confused with the sibling species, *M.weismanni*. Endemic in Thailand.	33
120	*Moinodaphniamacleayi* (King, 1853)	d, f, l, mi, po, ps, r, re, rf, sw	NE, W, C, S	Circumtropical	*Moinasubmucronata* and *Moinodaphniamacleayi* in Goulden (1968) are its junior synonyms.	1,3,6,11,12,13,18,19,20,30,34, 36,38
	Family Sididae					
121	*Diaphanosomacelebensis* Stingelin, 1900	l	S	Aus, Ori	It can be confused with *D.volzi* (Korovchinsky 1989). It is rare in Thailand.	30
122	*Diaphanosomadubium* Manuilova, 1964	f, re, sw	N, NE, W, E	Ori, Pal		13,17,28,35,36
123	*Diaphanosomaexcisum* Sars, 1885	f, ff, l, m, p, ps, r, rc, re, rf, sw,	N, NE, W, C, S, E	Circumtropical		1,2,3,4,5,6,8,11,12,13,17,18,19,20, 28,30,34,35,38
124	*Diaphanosomaelongatum* Korovchinsky & Sanoamuang, 2008*	d, l, r	N, NE, W, E, C	Ori	Endemic in Thailand.	17,28,36
125	*Diaphanosomamacrophthalma* Korovchinsky & Mirabdullaev, 1995	l, p, r, re, sw	NE	Ori		17
126	Diaphanosomacf.modigliani Richard, 1894	ml, p, re	C	Ori	It is rare in Thailand.	1,34
127	Diaphanosomasarsi Richard, 1894	ff, l, p, st, sw	N, NE, W, E, C, S	Circumtropical		1,3,4,8,11,12,17,28,36
128	*Diaphanosomasenegal* Gauthier, 1951	p, rc, rf, tp	N, NE, W, E	Afr, Ori	It is rare in Thailand. Korovchinsky and Sanoamuang (2008b) confirms that specimens found in Thailand are a subspecies, *D.senegalisanensis*.	17,28
129	*Diaphanosomatropicum* Korovchinsky, 1998	st	NE	Ori	It is rare in Thailand.	17
130	*Diaphanosomavolzi* Stingelin, 1905*	c, l, m, p, r, sw	N, NE, W, E, C, S	Afr, Aus, Ori		1,3,4,6,13,17,28,36
131	*Latonopsisaustralis* Sars, 1888	c, d, f, l, m, mi, p, po, ps, r, re, rf, sf, sw	N, NE, W, E, C, S	Afr, Aus, Neo, Nea, Ori		1,3,4,6,8,11,12,13,17,19,28,30, 36,38
132	*Pseudosidabidentata* Herrick, 1884	f, ff, l, p, rc, rf, st, sw	NE, W, C, S	Afr, Aus, Neo, Nea, Ori	It can be confused with *P.szalayi* (Chatteerjee et al. 2013).	1,3,6,8,11,12,13,19,20,30
133	*Pseudosidaramosa* (Daday, 1904)	p, ps, sw	NE, S	Aus, Neo, Ori		3,11,12
134	*Pseudosidaszalayi* (Daday, 1898)	l, rf, sf	N, NE, W, E	Ori, Pal	It is the closest species to *P.bidentata* (Korovchinsky 2010).	17,28,36,38
135	*Sarsilatonapapuana* Daday, 1900	ps	S	Ori	It is rare in Thailand.	22
136	*Sarsilatonaserricauda* (Sars, 1901)	p, rf	S	Neo, Nea, Ori, Pal		6,19
137	*Sidacrystallina* (O.F. Müller, 1776)	f, l, ps, rf	NE, S	Aus, Neo, Ori, Pal		4,11,12,13,19
138	*Sidaortiva* Korovchinsky, 1979	c, l, m, re, sw	NE, E	Ori, Pal	Previously recorded as *Sidacrystallinaortiva* (Tiang-nga et al. 2020) which it is the junior synonym.	17,36
