# Supplementary material for: Diversity and distribution of the cladocerans (Crustacea, Branchiopoda) in Thailand
Source: Biodivers Data J. 2023 May 25;11:e103553. doi: 10.3897/BDJ.11.e103553 (PMC10236201; doi:10.3897/BDJ.11.e103553)
Supplement: Supplementary material 1 — Definitions of each type of habitat in this study [file bdj-11-e103553-s001.docx]

**Definitions of each type of habitat in this study**

**Canal:** an artificial channel that is constructed to carry water to the fields to perform irrigation.

**Dam:** a wall built across a river that stops the river's flow and collects the water

**Estuary:** the brackish water area where fresh water from the river meets the salty open sea

**Floodplain:** the flatlands near a river and stream that regularly flooded

**Fish field:** fish farm pond

**Lake:** the large and rather deep natural wetland covered with diverse macrophytes

**Marsh:** an area of low flat ground that is always wet and soft, that often has grasses or reeds growing in it but no trees

**Mine:** waterlogged holes from mining

**Man-made lake:** the artificial tank for water storage and use in irrigation.

**Pond:** a body of standing water, either natural or artificial, that is usually very small when compared to a lake.

**Pool:** a small body of standing water

**Peat swamp:** flatland waterlogged area, where there is accumulation of peat and usually humic-brown water.

**River:** a natural wide flow of fresh water across the land into the sea, a lake, or another river.

**Roadside canal:** the artificial waterway along the road

**Reservoir:** a large natural or artificial water bodies used as a source of water supply.

**Rice field:** cultivated land parcels prepared for rice production, consisting of periodically flooded flat surfaces with irrigation channels or with rainfall.

**Saline rice field:** rice fields in the saline soil area, where water salinity range between 1.40 and 1.80 ppt.

**Stream:** the waterway run off the mountain through the river

**Swamp:** wetlands that are characterized by standing water and dense vegetation and that often have trees growing in them

**Temporary pond:** a shallow water bodies where encountered a periodic cycle of flooding and drought.

**Wastewater treatment pond:** The artificial ponds are designed to treat wastewater through the interaction of sunlight, bacteria, and algae.

**Waterfall:** water, especially from a river or stream, dropping from a higher to a lower point.
